# SUBJECTIVE COGNITIVE DECLINE MODERATES THE ASSOCIATION BETWEEN AGE AND MEMORY DECLINE IN MIDLIFE

**DOI:** 10.1093/geroni/igad104.3605

**Published:** 2023-12-21

**Authors:** Kyoung Shin Park, Jennifer Etnier, Samantha DuBois, Samuel Kibildis, Hadassah Som-Pimpong, Jarod Vance, Christopher Wahlheim

**Affiliations:** University of North Carolina at Greensboro, Greensboro, North Carolina, United States; University of North Carolina at Greensboro, Greensboro, North Carolina, United States; University of North Carolina at Greensboro, Greensboro, North Carolina, United States; University of North Carolina at Greensboro, Greensboro, North Carolina, United States; University of North Carolina at Greensboro, Greensboro, North Carolina, United States; North Carolina Institute for Medical Research, Inc, Durham, North Carolina, United States; University of North Carolina at Greensboro, Greensboro, North Carolina, United States

## Abstract

Subjective cognitive decline (SCD) represents self-perceived worsening of memory and thinking abilities, in the absence of objective cognitive impairment, and serves as a prognostic marker of Alzheimer’s disease (AD). However, no study to date has investigated whether SCD moderates the relationship between aging and memory before older ages. We tested the hypothesis that SCD interacts with age to predict memory performance in midlife. A subset of 123 cognitively unimpaired, middle-aged adults (Mage=56.6±6.44 years; 87% females) from an ongoing clinical trial (NIH R01AG058919) were included for analyses. Multiple linear regression models tested the effects of age and SCD in predicting performance of mnemonic discrimination, auditory-verbal memory, visuospatial memory, paired associative memory, and verbal logical memory, while controlling relevant covariates (e.g., education, sex, and APOE ε4). Based on the optimal cutoff of the Everyday Cognition Scale, 28.2% of the sample were considered to have SCD and 71.8% perceived themselves to have normal cognition. After covariate adjustments, there were significant main and interaction effects of SCD and age on mnemonic discrimination performance on the Mnemonic Similarity Task (MST) but not on other memory outcomes. The negative relationship between age and mnemonic discrimination was evident in middle-aged adults who self-report cognitive concerns but not in those without such concerns. Since mnemonic discrimination is highly sensitive to hippocampal function, these findings support the utility of the MST to detect age-related hippocampal changes and ensuing memory declines in midlife as well as the importance of early interventions among middle-aged adults with SCD to prevent AD.

